# The Impact of a Consecutive Process of Pulsed Electric Field, Sous-Vide Cooking, and Reheating on the Properties of Beef Semitendinosus Muscle

**DOI:** 10.3390/foods9111674

**Published:** 2020-11-16

**Authors:** Se-Ho Jeong, Eui-Chan Kim, Dong-Un Lee

**Affiliations:** Department of Food Science & Technology, Chung-Ang University, Anseong 17546, Korea; calvin0223@naver.com (S.-H.J.); rladmlcks2@naver.com (E.-C.K.)

**Keywords:** meat, pulsed electric field, sous-vide cooking, tenderization, texture property

## Abstract

The effects of a consecutive process of pulsed electric field (PEF) treatment, sous-vide cooking, and reheating on the properties of beef semitendinosus muscle were investigated. Fresh meats were PEF-treated with different electric field strengths of 1.0, 1.5, and 2.0 kV/cm, and then the control and PEF-pretreated beef samples were sous-vide cooked at 60 °C for up to 24 h. The PEF pretreatment resulted in tenderization of the fresh meat proportional to the increase in the electric field strength. A significant decrease in cutting force (by 35%) was observed after PEF treatment at 2.0 kV/cm. The hardness and chewiness of the meat were also significantly reduced by PEF treatment. After sous-vide cooking, the PEF-pretreated samples exhibited a significantly reduced cutting force, redness value (a^*^), and myoglobin content (mg/g) (*p* < 0.05). However, there were no significant differences in cooking loss and drip loss (*p* > 0.05). When the sous-vide-cooked meats were reheated in an oven (230 °C, 5 min), the reduced cutting force induced by the PEF pretreatment was retained.

## 1. Introduction

The term “sous-vide” means “under vacuum” in French and is defined as raw materials or raw materials with intermediate foods that are cooked under controlled conditions of temperature and time inside a heat-stable, vacuumized pouch [1]. The sous-vide method is a low temperature, long time treatment that inactivates microbial organisms in beef when heated above 60 °C, and prevents recontamination though use of the vacuumed packaging [2]. A one-step sous-vide cooking procedure is a popular food-service technique for meat with a high proportion of connective tissue, as moist cooking at higher temperatures can have a significant tenderizing effect though collagen gelation [3].

There have been some attempts to combine non-thermal processes, such as ultrasound [4], gamma irradiation [5], and electron beams [6], with sous-vide cooking to improve the quality of the sous-vide-cooked meats. A combination of high hydrostatic pressure and sous-vide cooking was investigated for the production of high-quality seafood [7], cowpea puree [8], and beef [9].

Pulsed electric field (PEF) technology has been adopted by the food industry due to its ability to control food spoilage, enhance mass transfer, and alter the textural properties while maintaining the quality of fresh foods. In industrial operations, PEF is commonly operated at a field strength of 10 kV/cm or higher for non-thermal pasteurization of liquid foods, or at 1~5 kV/cm to improve the mass transfer and alter the textural properties of food materials. Examples of low-field-strength applications include the use of PEF pretreatment for frying potatoes [10], PEF-assisted extraction of anthocyanin [11], PEF-assisted extraction of polyphenols [12], and accelerated dehydration of vegetables through increased mass transfer achieved via PEF pretreatment [13,14].

Several studies have evaluated the effects of PEF treatments on the quality characteristics of meat. Texture and color changes in PEF-treated meat have been widely investigated due to their strong impact on consumer acceptance [15,16]. Tenderization can be explained through the proteolysis of meat muscle tissue, and it has been shown that PEF treatment resulted in the proteolysis of troponin-T in meat muscle [16,17]. PEF treatment accelerates the denaturation and heat solubilization of connective tissue due to the electroporation effects [18]. The electroporation effect of the PEF treatment induces increased mass transfer of ions and other small molecules through the cell membrane, which can be applied to accelerate the meat drying and curing process.

Sous-vide cooking is a relative complex process since it includes both low-temperature vacuum heating and a high-temperature roasting process. Furthermore, sous vide is a time-consuming process, limiting its application to industrial processes. Therefore, it is required to reduce the sous-vide cooking time without changing the quality of the meat. The properties of PEF treatment meet these requirements. There has been some research on combining PEF and sous-vide techniques [19,20]; however, these studies were conducted only on sous-vide-cooked meat. A complete process for sous-vide-cooked meat should include a reheating or roasting process to create surface clusters. It would be of particular benefit to the food industry if the PEF pretreatment of meat can be shown to modify meat properties and reduce the sous-vide cooking time. The objective of this research was to evaluate the impact of PEF pretreatment on the selected physicochemical properties of raw- and sous-vide-cooked meat, and subsequently reheated meat.

## 2. Materials and Methods

### 2.1. Experimental Design

**Figure 1:**
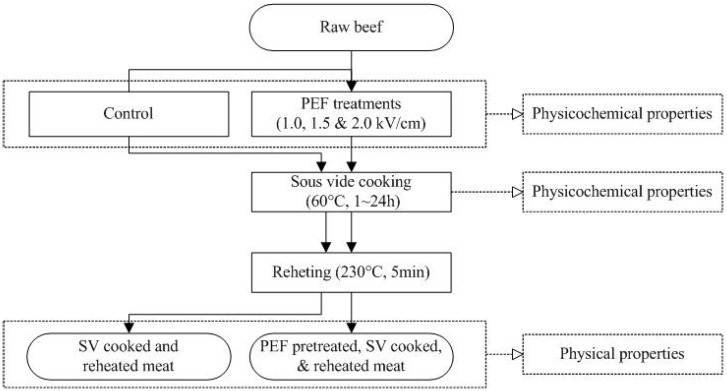
The process flow for the pulsed electric field (PEF) treatment, sous-vide cooking, and reheating.

Fresh raw beefs from the semitendinosus muscle were PEF-treated in triplicate at three PEF levels of 1.0, 1.5, and 2.0 kV/cm (Figure 1). The effects of the PEF pretreatment on the raw meat were evaluated by physicochemical analyses, including assessment of the textural properties, color, weight loss, drip loss, and Z-index. Subsequently, the untreated control and PEF-pretreated samples were vacuum packaged and sous-vide cooked at 60 °C for 24 h. The changes during sous-vide cooking were monitored by assessing the textural properties, color, myoglobin content, and cooking loss. Finally, the PEF-pretreated and sous-vide-cooked meats were reheated at 230 °C for 5 min to replicate standard restaurant preparation, and the textural properties were compared.

### 2.2. Samples

Beef semitendinosus muscle obtained from steers within 48 h of slaughter was used in this study. The steers were raised in the same region and purchased at a livestock wholesale market (Eumseong-gun, Korea). Two masses of semitendinosus muscle obtained from one individual steer were used for each experiment. All semitendinosus muscles used in this experiment were classified as “A+” grade. The fat and unnecessary tissue in the meat were trimmed, and the raw samples were stored at 4 °C before use.

### 2.3. PEF Treatment

The PEF treatment was conducted using a 5-kW pulse generator (HVP-5; DIL, Quakenbrück, Germany), in a batch treatment chamber with parallel electrodes spaced 8 cm apart. The meat was cut into rectangles (6 cm × 3 cm × 3 cm) and immersed in the chamber with 300 mL of tap water. The PEF treatment was conducted at electrical field strengths of 1.0, 1.5, and 2.0 kV/cm, with a fixed pulse width of 20 μs, and 200 pulses in each treatment. The PEF treatment was conducted at room temperature, and the increase in temperature induced by the PEF treatment was less than 3 °C.

### 2.4. Sous-Vide Cooking

The sous-vide cooking process was conducted by immersing vacuum-packaged meat in a water bath at 60 °C. The sous-vide cooking time was set to 1, 3, 6, 12, and 24 h, respectively, and all experiments were performed in triplicate.

### 2.5. Reheating (Roasting of the Sous-Vide-Cooked Meat)

The reheating process was modeled on the process undertaken in a restaurant kitchen. The sous-vide-cooked meat was roasted using a preheated convection oven at 230 °C for 5 min. The central temperature of the reheated meat was measured to be approximately 70 °C.

### 2.6. Estimation of Cellular Damage Based on the Biological Conductivity Measurements

The biological electrical conductivity (σ, S/m) of the meat was measured using an LCR meter (LCR-8000G; Gwinstek, New Taipei City, Taiwan) to estimate the deformation of the muscle fiber structure by the PEF treatment. The untreated control and PEF-treated samples were cut into cubes (1 cm × 1 cm × 1 cm), and the electrical conductivity of the samples was measured across the range of 1 kHz to 2 MHz. The degree of muscle fiber deformation was expressed by the electrical conductivity disintegration index Z (Z-index), calculated using the following equation [21]:

(1) $$Z=\left(\sigma -\sigma_{i}\right)/\left(\sigma_{d}-\sigma_{i}\right)$$

where σ (S/m) is the electrical conductivity at a frequency of 1 kHz, and the subscripts *i* and *d* indicate the electrical conductivity values of intact and completely damaged meat, respectively. The value of σ_d_ was measured from meat thawed at room temperature after freezing for 24 h. In this equation, *Z* = 0 for intact tissue and *Z* = 1 for completely ruptured tissue.

### 2.7. Cutting Force and Texture Profile Analysis

Cutting force measurements (N) were conducted using a texture analyzer (TAHDi/500; Stable Micro Systems Ltd., Godalming, UK) and based on the maximum shear force (N). A Warner–Bratzler flat blade and a 50-kg power cell were used for the measurements. The meat sample was cut into 3 cm × 1 cm × 1 cm strips and then cut perpendicular to the muscle fiber direction [22], with the crosshead speed set at 4 mm/s. At least six repetitions of the cutting force measurements were conducted.

The texture profile was measured using a texture analyzer (TA-XT; Stable Micro Systems Ltd., Surrey, UK). For texture profile measurements, the meat was cut parallel to the muscle fiber direction to form a cube shape (1 cm × 1 cm × 1 cm), and the samples were axially compressed in the muscle fiber direction to 50% of their initial height in two cycles, at a pre-test speed of 10 mm/s, a test speed of 2 mm/s, and a post-test speed of 2 mm/s. The samples were subjected to a two-cycle compression test with a cylindrical probe (35 mm diameter, P/35) [23]. The power cell used was 5 kg. The texture parameters from the force deformation curve were expressed as hardness, springiness, cohesiveness, and chewiness.

### 2.8. Color

The color of the meat was determined based on the CIE L*, a*, and b* values, measured using a colorimeter (UltraScan Pro; HunterLab, Reston, VA, USA) on the cut cross sections. A standard white plate with L* = 97.49, a* = 0.13, and b* = 0.04 was used for calibration.

### 2.9. Total Myoglobin Content

The total myoglobin content (mg/g) of the meat was measured from the absorbance spectra of the myoglobin molecules in a phosphate-buffered saline (PBS) solution (Sigma Aldrich, St. Louis, MO, USA), as described by Trout [24]. Measurements were conducted using a spectrophotometer (Genesys 20; Thermo Scientific, Waltham, MA, USA). A mass of 2 g was taken from the center of the meat and homogenized in 10 mL of 0.04 M PBS. After cooling in iced water for 1 h, the solution was centrifuged at 10,000× *g* for 30 min. The supernatant was filtered with filter paper (Whatman® 173 No. 1; Whatman plc, UK) and topped up with 25 mL of PBS. The absorbance spectra were measured at 525 nm and 700 nm and the total myoglobin content was calculated using the following equation:

(2) $$\mathrm{Total}\;\mathrm{myoglobin}\;\left(\frac{\mathrm{mg}}{g}\right)=\;A_{525\;}-A_{700\;}\times 2.30\times Dilution\;factor$$

### 2.10. Cooking Loss and Drip Loss

The cooking loss was calculated by measuring the initial and final weight. The cooking loss equation is given by:

(3) $$\mathrm{Cooking}\;\mathrm{loss}\left(\%\right)=\frac{initial\;weight-final\;weight}{initial\;weight}\times 100$$

Drip loss measurements were conducted using a centrifugation method [25]. Meat was cut into 10-g pieces and covered with filter paper. The samples were centrifuged at 4 ℃ at 1000 rpm for 10 min. The drip loss equation is given by:

(4) $$\mathrm{Drip}\;\mathrm{loss}\left(\%\right)=\frac{initial\;weight-final\;weight}{initial\;weight}\times 100$$

### 2.11. Statistical Analysis

All experiments were performed at least in triplicate, and the results are expressed as the mean ± standard deviation. The effects of the PEF treatments and the sous-vide time on the meat’s physical and chemical properties were assessed through two-way analysis of variance (ANOVA) using SPSS software, version 20 (IBM Corp., Armonk, NY, USA). The effects of each factor were assessed through one-way ANOVA. The main effects and interactions of the PEF pretreatment and sous-vide cooking time were analyzed using two-way ANOVA and the effects of each factor were assessed through one-way ANOVA. The difference between means was compared using Duncan’s multiple range test. A *p-*value < 0.05 was considered statistically significant.

## 3. Results and Discussion

### 3.1. PEF Pretreatment of Raw Beef

#### 3.1.1. Effects of PEF Pretreatment on the Electrochemical Properties of Raw Meat

**Figure 2:**
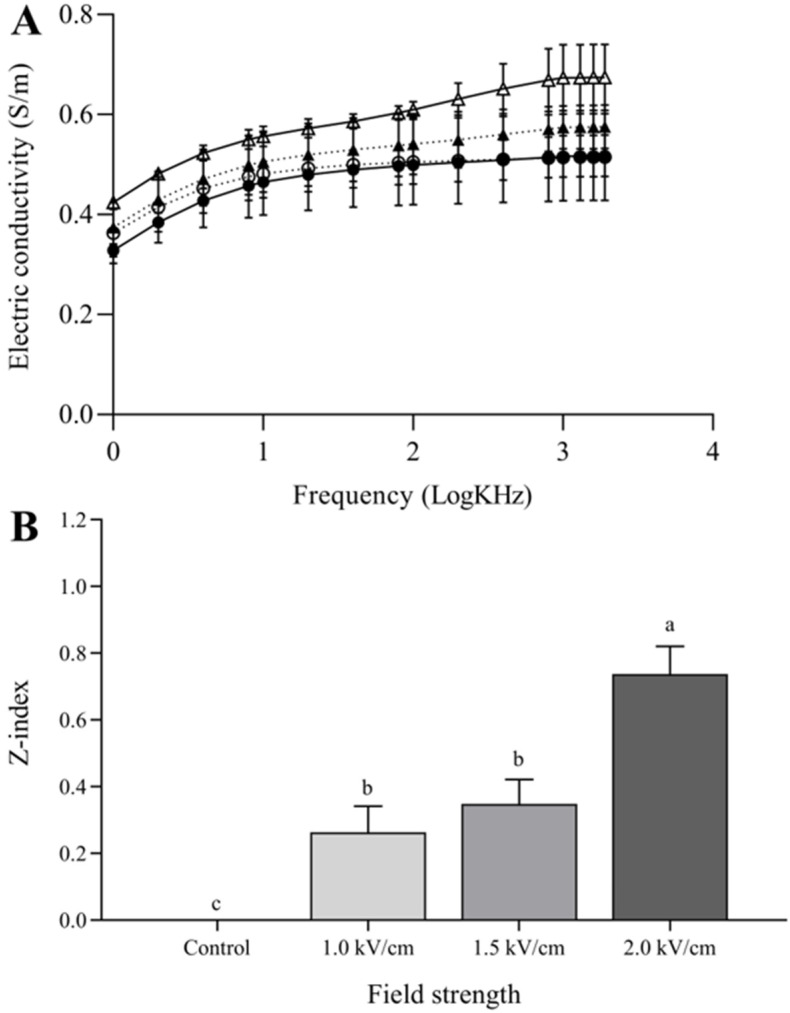
Electric conductivity spectra (**A**) and disintegration index, Z- index (**B**), of PEF-treated beef. The symbols in (**A**) are as follows: control (-●-); PEF with 1.0 kV/cm (-○-); PEF with 1.5 kV/cm (-▲-); PEF with 2.0 kV/cm (-△-). Bars represent the standard error of the mean (*n* = 3). Values are expressed as means ± standard deviation (*n* = 3). Means with different letters are significantly different between control and PEF treatments (*p* < 0.05).

The frequency-dependent electrical conductivity spectra of the control and PEF-treated beef samples were measured between 1 kHz and 1.9 MHz (Figure 2A). The lowest conductivity values were observed for the untreated control at all frequencies. The PEF treatment resulted in increased conductivity, and the increase in conductivity was dependent on the electric field strength. Figure 2B shows the Z-index obtained using Equation (1), with the electrical conductivity values measured at a frequency of 1 kHz. The Z-index is a conductivity-based method used to measure the degree of tissue disintegration in PEF-treated samples [21,26].

The electric conductivity and Z-index results indicated that the muscle tissue was deformed by PEF pretreatment. The PEF-treated beef exhibited increased electrical conductivity [27,28,29], and it has previously discussed that an increased conductivity in meat leads a change in membrane permeability [30].

#### 3.1.2. Effects of PEF Pretreatment on the Physical Properties of Raw Meat

Table 1 shows the effect of the PEF pretreatment on the physical properties of raw beef. The cutting force, hardness, and chewiness decreased proportionally as the field strength increased. A reduction in those properties were clearly observed at a PEF of 2.0 kV/cm. Under the PEF treatment at 2.0 kV/cm, the cutting force of the raw meat decreased significantly from 61.83 ± 8.72 to 40.34 ± 9.29 (*p* < 0.05). Similarly, the PEF treatment decreased the hardness and chewiness significantly from 11.70 ± 5.06 to 2.86 ± 2.03, and from 7.02 ± 3.22 to 1.57 ± 1.02, respectively (*p* < 0.05). Springiness and cohesiveness were not affected by the PEF treatment, and the PEF treatment did not change the other physical properties, including color. The water-holding property of meat was evaluated by weight loss and drip loss, and both were maintained under PEF treatment conditions (*p* > 0.05).

Previous findings of the effect of PEF on meat texture are controversial. One study showed that PEF treatment increased the shear force of meat from 79.3 N to 83.2 N [27]. Others reported that PEF pretreatment at 1.4 kV/cm showed a tendency towards reducing the toughness of beef samples, although the reduction was not significant [22]. However, the tenderization by PEF treatment has been observed and explained by degradation of troponin-T [16].

### 3.2. Sous-Vide Cooking of PEF-Pretreated Beef

#### 3.2.1. Changes in Cutting Force in the Sous-Vide Cooked Beef

Table 2 shows the changes in the cutting force in the control and PEF-pretreated meat after a different sous-vide cooking time. Comparisons between groups due to individual factors were based on one-way ANOVA. The PEF pretreatment affected the cutting force of the sous-vide-cooked meat, and clear differences were observed between the control and PEF pretreatment at 2.0 kV/cm. There were significant reductions in cutting force between the PEF 2.0 kV/cm samples and the control at all sous-vide cooking times. The PEF pretreatment at 1.0 and 1.5 kV/cm showed intermediate characteristics; the PEF pretreatment at 1.0 kV/cm exhibited properties more similar to the control; and the PEF pretreatment at 1.5 kV/cm exhibited characteristics between the control and PEF at 2.0 kV/cm. The interaction effect of the PEF treatment and sous-vide time was shown (*p* < 0.01). This means that an increased tenderness was observed as the field strength and sous-vide cooking time are increased.

The reason for setting the sous-vide temperature at 60 °C is that sous-vide cooking is generally processed between 60 °C and 70 °C and the meat connective tissue contracts when it is cooked over 65 °C [31]. Since all samples were cooked without muscle contraction, we consider that the tenderization effect of the PEF pretreatment was retained during sous-vide cooking, especially for the PEF pretreatment at 2.0 kV/cm where the tenderization effect was greatest.

#### 3.2.2. Changes in Total Myoglobin Content, Red Color, and Cooking Loss in Sous-Vide-Cooked Beef

**Figure 3:**
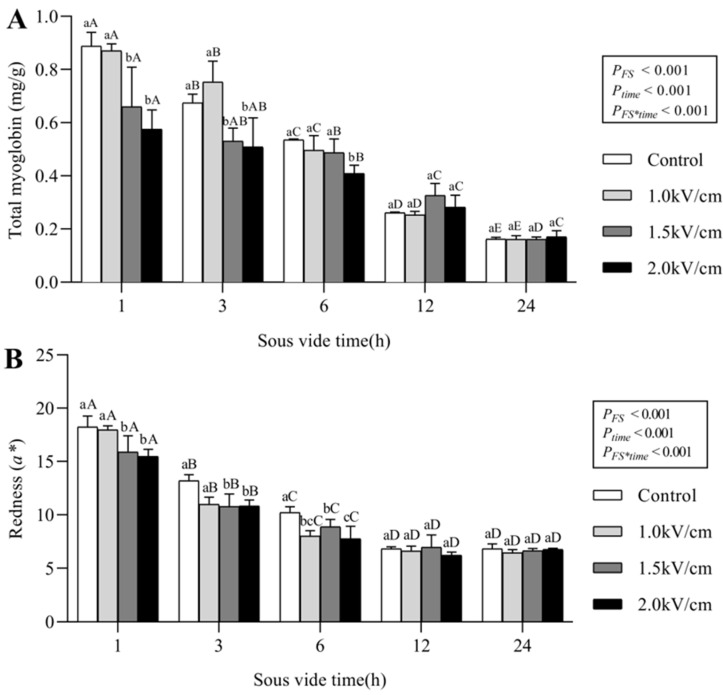
Changes in total myoglobin content (**A**) and redness (**B**) in the control and PEF-pretreated beefs by sous-vide cooking (60 ℃, 1 h to 24 h). *P_FS_*, *P_time_*, and P_FS*time_. were based on two-way ANOVA. Different lowercase letters (**a**,**b**) in the same group indicate significant differences regarding field strength (*p* < 0.05). Different uppercase letters (**A**–**D**) in the same color bar indicate significant differences regarding sous-vide time (*p* < 0.05). The letters were based on one-way ANOVA; post-hoc analysis was conducted using Duncan’s multiple range test.

Figure 3 shows the total myoglobin content and redness of the sous-vide-cooked beef. Myoglobin, including oxy-myoglobin and met-myoglobin, is converted into met-myochromogen when meat is completely cooked. As the amount of myoglobin decreases, the red color of the meat changes to a pale gray color. The difference in total myoglobin content between the control and PEF-pretreated meat was clear in the early stage of sous-vide cooking, but the difference became statistically non-significant after 12 h of sous-vide cooking (*p* < 0.05). This phenomenon was also observed for the redness of the sous-vide-cooked meat. The values of b* reduced significantly as the sous-vide cooking time increased, and there was no significant difference between the control and PEF-pretreated samples after 12 h of sous-vide cooking.

**Figure 4:**
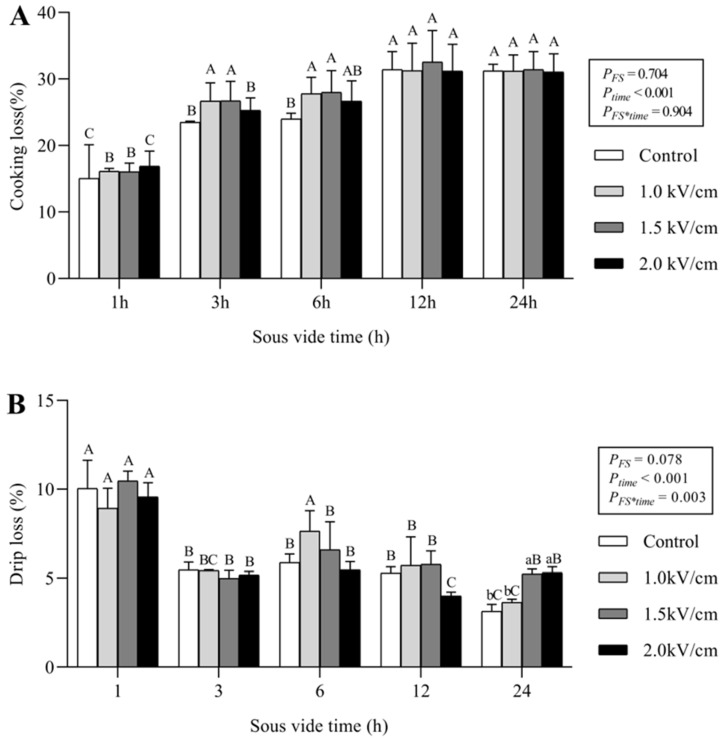
Changes in cooking loss (**A**) and drip loss (**B**) in the control and PEF-pretreated beefs by sous-vide cooking (60 °C, 1 h to 24 h). *P_FS_*, *P_time_*, and P_FS*time__._ were based on two-way ANOVA. Different lowercase letters (**a**,**b**) in the same group indicate significant differences regarding field strength (*p* < 0.05). Different uppercase letters (**A**–**C**) in the same color bar indicate significant differences regarding sous-vide time (*p* < 0.05). The letters were based on one-way ANOVA; post-hoc analysis was conducted using Duncan’s multiple range test.

Figure 4 shows the cooking loss and drip loss of the sous-vide-cooked beef. Cooking loss was affected by sous-vide time (*p* < 0.001), although the effect of field strength and the interaction effect were not significant (*p* = 0.704 and 0.904, respectively). Cooking loss increased significantly with a longer sous-vide time for all samples. After 12 h of sous-vide cooking, no significant difference was observed between the control and the PEF-pretreated samples (*p* > 0.05). These cooking loss results are similar to those discussed in other studies, in which the cooking loss reached a specific level at longer cooking times [19,32].

Drip loss was affected by sous-vide time (*p* < 0.001), but not by PEF pretreatment (Figure 4B). Drip loss showed a maximum value in the samples that were sous-vide cooked for 1 h and decreased continually with an increase in sous-vide cooking time. Drip loss represents the water-holding capacity of meat, and an increase in drip loss indicates a decrease in the water-holding capacity. It has been shown that PEF treatment does not affect the drip loss of meat [27,33]. Drip loss is usually measured in raw meat. However, PEF treatments did not cook the meat at all, and sous-vide cooking is also regarded as very gentle cooking method. Therefore, we measured the drip loss to determine the possible additional meat juice loss during the sous-vide cooking process due to PEF treatments.

Generally, changes in meat properties after PEF pretreatment were field-strength dependent, and the PEF-induced changes were retained after a sous-vide treatment time of 6 h. Further sous-vide cooking masked the effects of the PEF on the meat properties, and the difference between the control and the PEF-pretreated samples decreased. Therefore, the sous-vide cooking time was fixed at 6 h for subsequent experiments.

### 3.3. Reheating of the Sous-Vide Cooked Beef and Impact on Cutting Force

**Figure 5:**
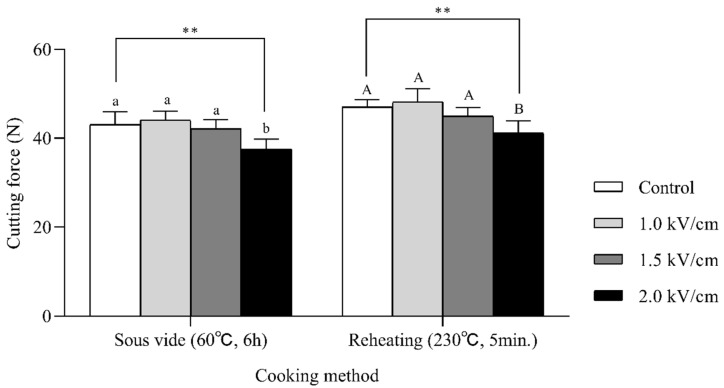
Cutting force of the sous-vide-cooked beefs (60 °C, 6 h) and reheated beefs (230 °C, 5min.) after PEF pretreatment. Sous-vide cooking was conducted at 60 °C and for 6 h; reheating was conducted at 230 °C and for 5 min after the sous-vide cooking. All values are expressed as the mean ± SD (*n* = 6). Different letters in the same group indicate a significant difference between control and PEF treatments (*p* < 0.05). The letters were based on one-way ANOVA; post-hoc analysis was conducted using Duncan’s multiple range test. ** *p* < 0.01.

Sous-vide-cooked meat is usually reheated at its surface to improve the flavor, texture, and taste. Therefore, we investigated the effect of reheating on the cutting force of sous-vide-cooked meat. Figure 5 shows the cutting force of the sous-vide-cooked beef and sous-vide-cooked followed by oven-cooked beef. The sous-vide cooking was conducted at 60 °C for 6 h (see Section 3.2.2). The PEF pretreatment of 2.0 kV/cm resulted in a significantly reduced cutting force compared to the control after 6 h of sous-vide cooking. The cutting forces of the reheated beef samples were slightly increased due to the 290 formation of a surface crust and the drying effect. However, the difference between the control and the 2.0 kV/cm-pretreated sample remained after the reheating process. This result shows that the effects of PEF treatment are retained after sous-vide cooking and reheating. There have been some studies of sous-vide-cooked meat; however, these did not include the reheating process [19,23,34].

## 4. Conclusions

Application of PEF treatment with different field strengths (1.0, 1.5, and 2.0 kV/cm) significantly influenced the textural properties of raw meat. The cutting force, hardness, and chewiness of the meat decreased proportionally as the field strength increased. The field strength dependence of these structural changes was confirmed by the Z-index obtained from the frequency-dependent electrical conductivity spectra. The differences in texture were not a result of changes in the water-holding capacities of the meat since they were not affected by the PEF treatment. The untreated control and PEF-treated samples were sous-vide cooked at 60 °C for 1, 3, 6, 12, and 24 h, and the impact of sous-vide cooking on the physicochemical properties of the meat were evaluated. Sous-vide cooking significantly reduced the total myoglobin content, red color, and drip loss of meat with increasing sous-vide cooking time, while minimal changes in the cutting force were observed. PEF-induced changes were retained with a sous-vide treatment time of 6 h, while further sous-vide cooking masked the effect of the PEF treatment on the meat and reduced the difference between the control and PEF-pretreated samples. We investigated the effect of reheating on the cutting force of the sous-vide-cooked meat. The cutting forces of the reheated beef samples increased slightly after reheating due to the formation of a surface crust and the drying effect. However, the tenderization effect by the 2.0 kV/cm-pretreated sample was retained after sous-vide cooking (60 °C, 6 h) and the reheating process (230 °C, 5 min). The effects of PEF pretreatment followed by sous-vide cooking and reheating on meat properties have not been previously reported, and these findings have important implications for the food industry and catering companies working with value-added meat products.

## Figures and Tables

**Table 1:** The texture, color, and water-holding properties of PEF-treated raw meat.

Parameters	Field Strength			
	Control	1.0 kV/cm	1.5 kV/cm	2.0 kV/cm
Textural Properties				
Cutting force (*N*)	61.83 ± 8.72 ^a^	53.24 ± 7.05 ^ab^	48.13 ± 7.73 ^bc^	40.34 ± 9.29 ^c^
Hardness (*N*)	11.70 ± 5.06 ^a^	9.74 ± 3.78 ^a^	8.51 ± 2.27 ^a^	2.86 ± 2.03 ^b^
Springiness	0.98 ± 0.01 ^a^	0.95 ± 0.07 ^a^	0.92 ± 0.04 ^a^	0.97 ± 0.03 ^a^
Cohesiveness	0.62 ± 0.03 ^a^	0.59 ± 0.03 ^a^	0.57 ± 0.03 ^a^	0.59 ± 0.0 ^a^
Chewiness (*N*)	7.02 ± 3.22 ^a^	5.54 ± 2.55 ^a^	4.52 ± 1.52 ^ab^	1.57 ± 1.02 ^b^
Color				
L*	41.22 ± 2.28 ^a^	40.39 ± 1.31 ^a^	39.58 ± 1.49 ^a^	40.80 ± 2.10 ^a^
a*	17.11 ± 1.01 ^a^	16.87 ± 1.74 ^a^	15.81 ± 0.58 ^a^	16.50 ± 1.08 ^a^
b*	13.83 ± 0.94 ^a^	13.74 ± 1.08 ^a^	13.61 ± 0.63 ^a^	12.83 ± 0.55 ^a^
Water-Holding Properties				
Weight loss (%)	-	0.16 ± 0.10 ^a^	0.18 ± 0.09 ^a^	0.28 ± 0.18 ^a^
Drip loss (%)	4.29 ± 0.82 ^a^	3.46 ± 0.80 ^a^	4.47 ± 0.36 ^a^	4.36 ± 0.28 ^a^

Values are expressed as the mean ± standard deviation (*n* = 6). Means with different letters are significantly different between control and PEF treatments (*p* < 0.05).

**Table 2:** Changes in the cutting force in the control and PEF-treated beefs after sous-vide treatment (60 °C, 1 h to 24 h).

Parameters	Field Strength	Sous-Vide Time				
		1 h	3 h	6 h	12 h	24 h
Cutting force (*N*)	Control	44.27 ± 5.06 ^aA^	39.79 ± 4.09 ^bA^	45.05 ± 2.18 ^aA^	40.74 ± 6.39 ^abA^	44.70 ± 5.34 ^aA^
	1.0 kV/cm	43.16 ± 4.69 ^aB^	50.17 ± 2.60 ^aA^	45.07 ± 3.11 ^aB^	42.86 ± 3.26 ^aB^	33.38 ± 2.31 ^bC^
	1.5 kV/cm	41.48 ± 2.18 ^abA^	38.56 ± 5.16 ^bAB^	38.99 ± 3.86 ^bAB^	35.16 ± 4.59 ^bB^	26.97 ± 2.46 ^cC^
	2.0 kV/cm	35.75 ± 4.02 ^bAB^	31.69 ± 3.91 ^cB^	40.51 ± 6.88 ^abA^	34.64 ± 5.99 ^bAB^	30.42 ± 2.57 ^bcB^
*p-*value	Field strength	*P_FS_* < 0.001	Sous-vide time	*P_time_* < 0.001	Interaction	P_FS*time_ < 0.001

Values are expressed as the mean ± standard deviation (*n* = 6). Values with different lowercase letters in the same cooking time indicate significant differences regarding the electric field strength (*p* < 0.05). Values with different uppercase letters in the same electric field strength indicate significant differences regarding the cooking time (*p* < 0.05). *P_FS_*, *P_time_*, and P_FS*time__._ were based on two-way ANOVA.
